# Using games to draw attention to urban nature exposure and its therapeutic benefits: a study protocol

**DOI:** 10.3389/fpubh.2026.1913334

**Published:** 2026-08-24

**Authors:** Joonsoo Sean Lyeo, Allison Williams

**Affiliations:** School of Earth, Environment and Society, McMaster University, Hamilton, ON, Canada

**Keywords:** mixed method, nature exposure, photovoice, therapeutic landscape, therapeutic landscape theory, urban nature

## Abstract

**Background:**

Nature exposure is an important determinant of physical, psychological, and social development and well-being. However, it has been suggested that today’s youth are less likely to engage with the natural world than generations before. This trend has been attributed, in part, to behavioral and attitudinal shifts exacerbated by the rise of sedentary lifestyles, digital hobbies, and nature disaffection. These factors have been especially pronounced for those living in urban areas. There may be an opportunity to counter this growing nature disconnection by promoting opposing behavioral and attitudinal shifts. Persuasive games offer a promising avenue for achieving this by leveraging game design to encourage nature exposure.

**Methods:**

The primary objective of this study will be to understand to what extent a mixed-media gaming intervention can be used to investigate urban nature exposure, while also drawing attention to the therapeutic benefits associated with urban nature exposure, in a population of university-attending youth (18–25 years old). Participants will be assigned to either a control group or a *City Safari* intervention group, the intervention comprising a persuasive game modeled after “bingo photography”. Participants in the intervention group will be tasked with taking pictures of their urban nature surroundings. A select number of participants from the intervention group will be invited to elaborate on their pictures in a semi-structured, one-on-one interview drawing from a modified photovoice methodology. The resultant transcripts will be analyzed through thematic analysis.

**Discussion:**

The findings from this project are expected to provide the basis for knowledge communication deliverables targeted to both traditional academic and general non-academic audiences.

## Background

Nature exposure is well-documented to be associated with wide-ranging benefits for health and well-being, including significant reductions in stress, anxiety, depression, cardiovascular mortality, and self-reported well-being (1–3). The association between nature exposure and well-being have been observed to fluctuate over time, with effects often accumulating over an individual’s life course (3). This suggests that early-life nature exposure may have sustained benefits for later-life mental health and well-being, pointing to the therapeutic benefits of enabling sustained nature exposure from a young age (3).

Despite the growing recognition of the therapeutic benefits of nature exposure, it has been suggested that the divide between the so-called human and natural worlds has only increased in recent years (4). For instance, Soga and Gaston (5) contend that many recent changes in human lifestyles—such as the increasing digitalization of social engagement, the growth of sedentary pastimes, and the overscheduling of our day-to-day lives—have resulted in an overall decreased interest in engaging with the natural world. The resulting alienation of humans from the natural world has been termed the “extinction of experience” (5, 6).

In the wider literature, there has been speculation on the extent to which youth residing in urban areas may be particularly susceptible to the extinction of experience (7). With more than half of the global population now living in cities, it has been suggested that youth will have their interactions with the natural world filtered through the lens of urban landscapes (7). Processes such as urban densification and the closure of third places have been said to contribute to a “nature deficit” by limiting access to nature (8). Per this argument, with fewer opportunities to readily experience nature, urban youth are more likely to experience nature exclusively in heavily curated settings, such as indoor houseplants and pets (7).

These concerns are especially pertinent given not only the well-documented therapeutic benefits of nature exposure, but also the detrimental impacts of certain urban environmental exposures on mental health and well-being (9). At the same time, it should be noted that despite the widespread conceptualization of an urban-nature dichotomy, urban environments are far from devoid of natural features (10). Parks, backyards, and green spaces harbor slices of biodiversity within the urban landscape. Despite this, it has been observed that urban residents tend to adhere to wholly artificial perceptions of what nature ought to look like (5, 6).

Because nature and urbanity are frequently seen as being opposed, urban residents are often primed to overlook their already-existing interactions with the comparatively mundane forms of nature found in cities (5, 6). This framing is perpetuated by the longstanding notion that humans are apart from, rather than a part of, the wider ecosystem (10). These framings can make it difficult for urban residents to notice and appreciate when nature exists on smaller, less-imposing scales (10). As urban residents rarely consciously interact with the nature in their immediate surroundings, urban nature is frequently taken for granted, backgrounded, or rendered invisible (5, 6).

Urban areas do not necessarily represent a space of complete disconnection from the natural world. Nevertheless, Rautio et al. (11) concede that although nature is physically available in most urban areas, it often goes underutilized and unnoticed. It still holds that, for many urban youth populations, the amount of time spent in nature is decreasing. Corroborating the previously mentioned findings of Soga and Gaston (5, 6), Rautio et al. (11) have attributed this underexposure of nature to behavioral and lifestyle changes, such as a growing preference for digital media over physical play spaces. These changes have, in turn, been abetted by the tightening of parental controls and the penetration of technology into the lives of urban youth.

Because this phenomenon appears to have been driven by recent changes in behaviors and lifestyles, there may be an opportunity to encourage nature exposure through diametrical behavioral changes (5, 6). The notion that population health outcomes can be bolstered in this manner has already been recognized by a burgeoning body of health promotion literature (12). Within this academic discourse, there has been renewed interest in understanding the mechanisms underlying behavioral change—particularly those concerning motivation (12).

Games have long been recognized as well-suited for harnessing motivation in pursuit of specific goals (13). This recognition reflects the understanding that games are, by their very nature, intrinsically motivating. The motivation for gaming often rests in the activity itself, as seen through the completion of in-game objectives, progression of in-game events, or competition against opponents in in-game competitions (13). To exploit the built-in appeal of games to influence player behavior, various fields—from psychology to economics—have already sought to either develop their own games or incorporate game mechanics in non-gaming contexts (14).

## Methods

### Research question

The primary research question addressed in this study will be: To what extent can the *City Safari* gaming intervention be used to investigate urban nature exposure—while emphasizing the therapeutic benefits associated with urban nature exposure—in a population of university-attending youth? We hypothesize that those assigned to take part in the *City Safari* gaming intervention will report greater improvements in certain measures of nature connection and subject health and wellbeing—described below—than those who do not take part.

### Study setting

Participants will be recruited from the student population of McMaster University, a post-secondary institution located in the City of Hamilton, Ontario, Canada. All data collection will be conducted within the City of Hamilton.

### Sampling and recruitment

The initial sampling strategy will comprise a combined convenience- and criterion-based approach. This initial sampling strategy will aim to facilitate the rapid recruitment of eligible participants. Participants will be considered eligible as long as they meet the following inclusion criteria: (1) they are between 18 and 25 years of age at the time of enrolment; (2) they are registered students at McMaster University; (3) they have access to a phone with picture-taking capabilities; and (4) they are comfortable with holding a conversation in English.

**Figure 1:**
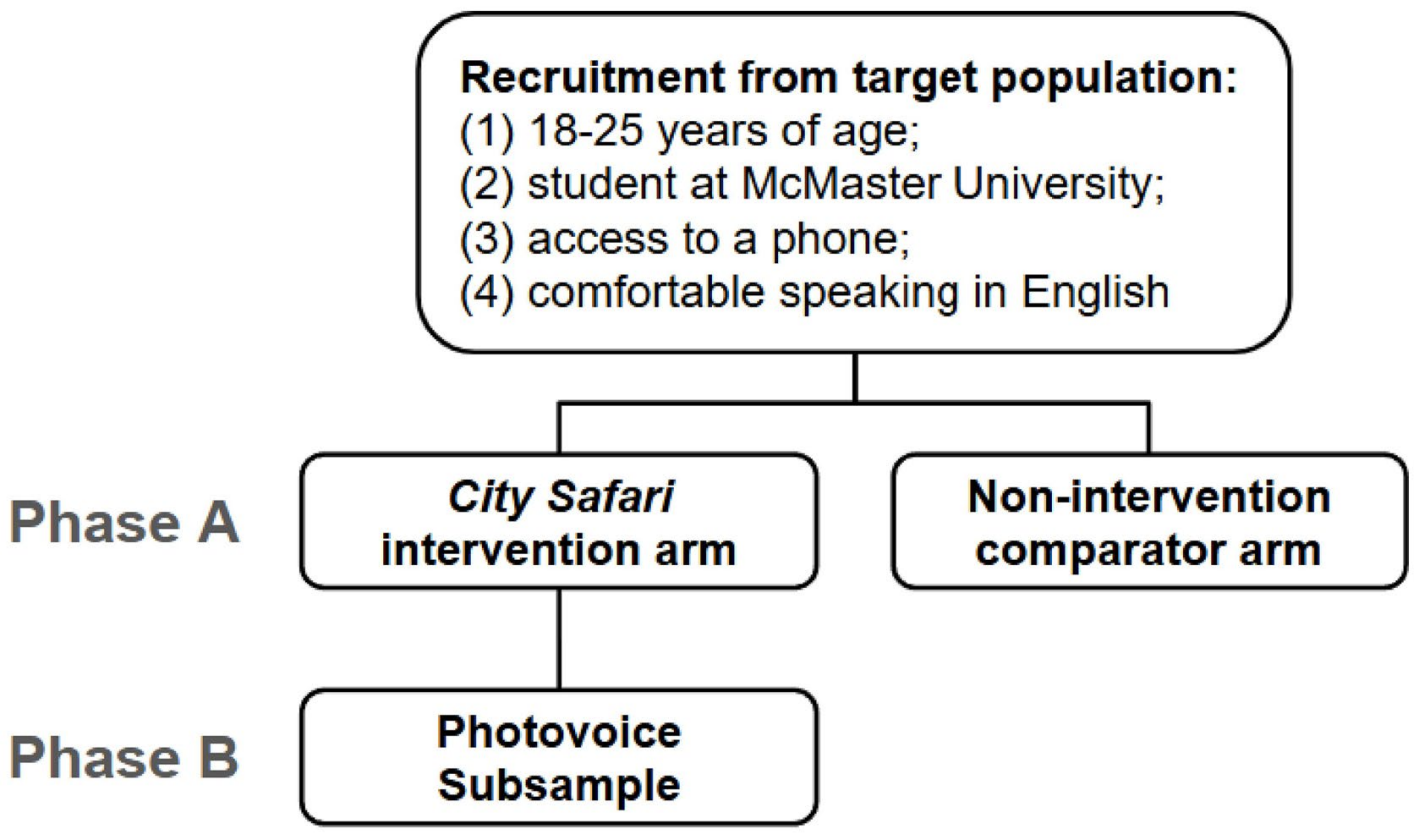
Summary of phases A and B.

The intention will be to run this study twice (as “Iteration A” and “Iteration B”), with each Iteration involving a different sample recruited from the same population. Each Iteration will consist of a “Phase A” and a “Phase B”. The sampling strategy discussed in the preceding paragraph will guide the first Phase of recruitment (Phase A) in each Iteration. A visual summary of the sampling process has been provided in Figure 1.

During Phase A, initial recruitment will consist of disseminating recruitment materials to as wide an eligible population as possible by posting physical posters, faculty mailing list email blasts, social media posts, and word-of-mouth communication. Participants recruited during Phase A will be randomly assigned to either a game-based intervention arm or a non-intervention comparator arm using simple random assignment generated by a computer. Using G*Power 3.1.9.1, it was calculated that, for Phase A, a minimum sample size of 48 participants (per arm) would be needed with an alpha of 0.1, a statistical power of 0.80, a medium-to-large effect size (Cohen’s f of 0.3), and a moderate pre-to-post correlation (*ρ*) of 0.5 (15). It should be acknowledged that any participants lost to follow-up during Phase A, who do not request their data to be withdrawn from the study, will have their incomplete responses handled through intent-to-treat analysis.

A subset of the participants assigned to the intervention arm during Phase A will be invited to participate in Phase B. The selection of participants for inclusion in Phase B will be guided by the theoretical sampling strategy outlined by Thorne (16). To this end, a comparative summary of the sociodemographic characteristics of the participants in the intervention arm will guide the targeted selection of Phase B participants. As per Thorne (16), the goal of theoretical sampling is to achieve maximal variation—i.e., to capture as wide a range of experiences as possible through the selection of participants who differ along key attributes within the target population. As such, when selecting participants for the Phase B subsample, their sociodemographic characteristics will be recursively assessed against those of the original intervention arm. The goal of this process is to ensure that the diversity of sociodemographic characteristics in the full sample will also be reflected in the Phase B subsample.

Furthermore, in contrast to Phase A, during which the goal will be to recruit as many eligible participants as possible, Phase B’s final sample size will be informed by considerations of “information power”, as outlined by Braun et al. (17) As per Braun et al. (17), the prevailing approach of “sampling until saturation” is at odds with the central ethos of reflexive thematic analysis, the qualitative analysis approach that will be employed in this study, which stipulates that there are infinite variations of the human experience. Information power provides an alternative method of delineating a desired sample size by accounting for the breadth of the guiding research question, the sample specificity, the presence of established theory, the anticipated quality of dialogue, and the intended analytical approach (18). Guided by this approach, Phase B will comprise a sample size of 15 to 20 participants.

### Intervention

As discussed, the same study methodology will be conducted twice, with each Iteration comprising a different pool of participants representing the same population. The two Iterations of the study methodology will adhere to the same sample strategy (as seen in Phase A and Phase B) and will use the same set of materials for data collection. They will differ only in their time of administration. The first Iteration will be conducted during the spring/summer semester of 2026 (May to August 2026), while the second Iteration will be conducted during the fall semester of 2026 (September to December 2026). Each Iteration will be conducted in a different season, with the first taking place in the summer and the second in the fall. The data collected from each Iteration will be analyzed as part of separate ANCOVA models.

In each Iteration, participants assigned to the intervention arm during Phase A will be invited to spend 2 weeks playing *City Safari*, a game modeled after bingo photography. Gameplay will consist of both analog and digital components, with participants receiving printouts of the bingo sheets (analog) and completing objectives using the cameras on their personal devices (digital). A mixed-media format was chosen, in part, to address concerns regarding the consumption of digital media as a significant risk factor for the disconnection of youth from the natural world (10). This concern has been exacerbated by evidence of deleterious health outcomes associated with excessive digital media consumption, including digital media addiction, reduced attentivity, and impaired emotional processing (19). Given that most gaming interventions for motivating nature exposure have, to date, relied on entirely digital delivery methods, this study presents an opportunity to assess the impact of a mixed analog-and-digital approach.

**Figure 2:**
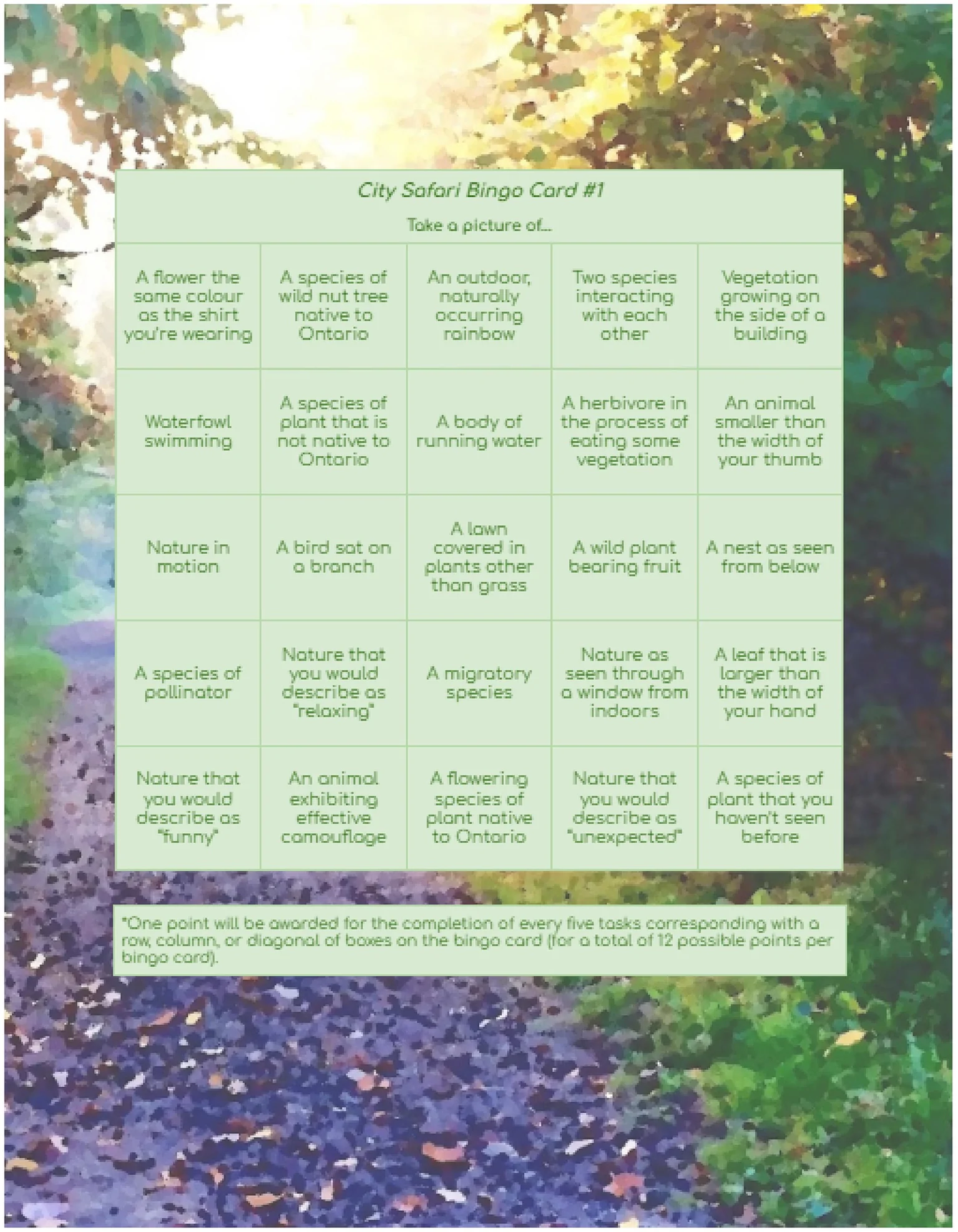
Sample *City Safari* bingo sheet.

The participants assigned to play *City Safari* during Phase A will be asked to complete their tasks independently of other participants. Participants assigned to the intervention arm will receive five bingo cards. Each card will consist of a five-by-five grid of squares for a total of twenty-five squares per card. Each square will outline a task requiring the participant to photograph themselves interacting with the natural features of their urban surroundings. A sample *City Safari* bingo sheet has been provided in Figure 2.

Due to the variability in how “urban nature” is defined in the extant literature—especially as it pertains to the dubiousness of the human-nature interface—for this study, urban nature will be defined as spatial features falling into one of the three categories of “mundane nature” highlighted by Hoyle (20). These categories are: (1) remnant nature, comprising pre-existing wilderness that has defied urban development; (2) cultivated nature, comprising nature that has been integrated into built urban infrastructure; and (3) nature on display, comprising settings that have been intentionally designed to enable heavily curated human-nature contact.

**Figure 3:**
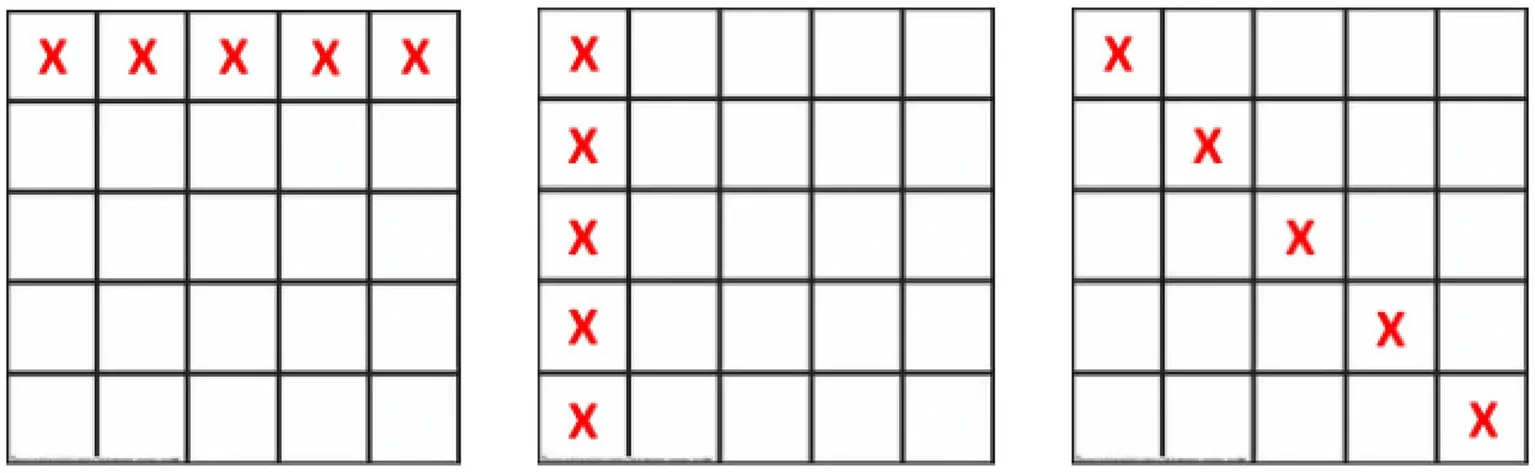
Examples of valid, point-earning rows, columns, and diagonals.

Participants will receive one point for each five-square row, column, or diagonal that they claim on a bingo card. To claim a square, participants must take a photograph demonstrating that they have met the criteria outlined on each square’s corresponding task (Figure 3). As there are twelve possible rows, columns, or diagonals per *City Safari* bingo card—and five *City Safari* bingo cards in total—participants may score a maximum of 60 points during Phase A.

**Figure 4:**
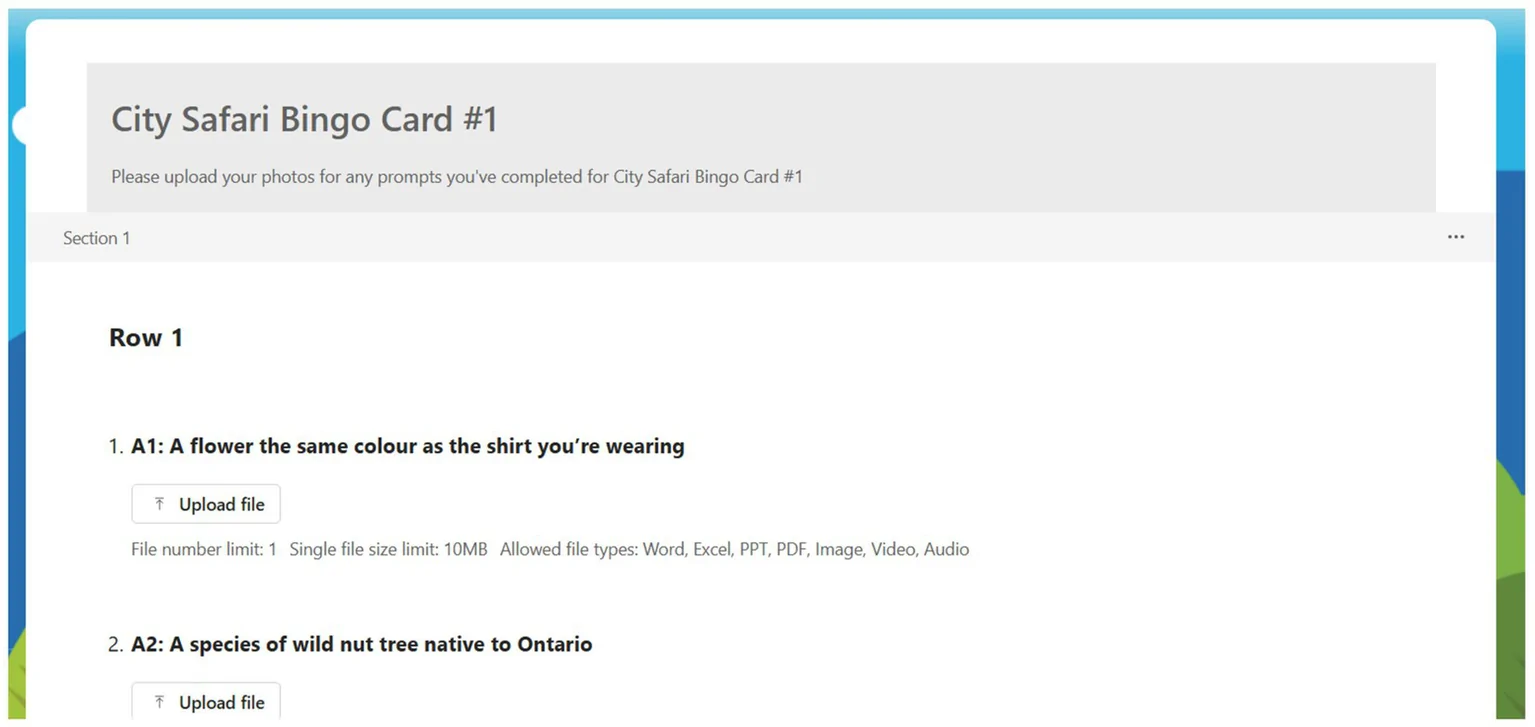
*City safari* photograph submission portal.

Participants will be instructed to submit their photographs to the researcher via a submission portal, provided in Figure 4. Each submitted photograph will be reviewed by the lead researcher, who will verify its integrity and validity. To assess a photograph’s integrity, the researcher will check its metadata to ensure that it was taken with the City of Hamilton using a digital device. To verify a photograph’s validity, the researcher will qualitatively assess its contents to review whether it meets the criteria outlined in the task to which it has been submitted. Any photographs found to deviate from these assessments of integrity and validity will be removed from analysis.

Participants assigned to the non-intervention comparator arm will not be invited to participate in *City Safari* during the intervention period. However, at the end of the intervention period, following the second stage of data collection, participants from the non-intervention comparator arm may opt to receive a copy of the *City Safari* game materials for their own personal use and reference.

Participants assigned to each arm, both the intervention and non-intervention arms, will be entered into a raffle for one of five $50 gift cards. In total, ten gift cards will be distributed to participants—five to those in the intervention arm and the remaining five to those in the non-intervention arm. The winners of each raffle will be revealed at the end of the data collection period. Entry into the gift card raffle will serve as extrinsic motivation, enticing enrolment into the study itself. In contrast, only participants in the intervention arm will be exposed to the *City Safari* gameplay, with the gameplay itself intended to serve as motivation for greater nature exposure.

### Prototyping

In line with the overarching goal of this study, each task listed on the *City Safari* bingo card will be designed to incentivize participants to engage with urban nature. To this end, the tasks will need to be created to encourage mindful attentiveness to the natural features that comprise everyday urban geographies. Furthermore, these tasks must be designed to ensure that they are feasible to accomplish within the prespecified geographic setting. Care must also be taken to ensure that each task can be completed safely and in an accessible manner, and that participants can reasonably complete them without placing themselves in positions that could cause harm or discomfort. To this end, tasks should be designed to be achievable by a wide range of individuals with varying physical and mental abilities.

Before data collection and the administration of the *City Safari* intervention, a game prototype will be developed to solicit feedback on the aforementioned features. This game prototype will be presented to two focus groups, each comprising four to five participants enrolled through targeted recruitment. This targeted recruitment will be directed at the leadership and general membership of community organizations, student clubs, government and non-government organizations, and neighborhood communities with an expressed mandate to encourage positive human-nature experiences. Focus group members will be selected to maximize variation along gender, ethnicity, and lived experience. Any individuals who choose to participate in the focus group will not be eligible for inclusion in the main study.

The focus groups will be asked to provide feedback on certain elements of the *City Safari* game design, including their thoughts on the tasks listed on each bingo card, the visual esthetics, and their impression of the core gameplay mechanics. During the focus groups, participants will be asked about the extent to which the tasks included in the *City Safari* prototype are achievable, accessible, enjoyable, and relevant to the goal of fostering engagement with urban nature. The feedback provided will be used to refine the prototype into a fully realized final version, which will serve as the basis for the *City Safari* intervention.

### Data collection

The data collection methodologies will differ across Phases A and B. At the beginning of Phase A, all participants, regardless of whether they had been assigned to the intervention arm or the non-intervention comparator arm, will be asked to complete a sociodemographic questionnaire. The sociodemographic questionnaires will be issued immediately after each participant’s assignment to a study arm. The sociodemographic questionnaire will be used to collect baseline data on potentially confounding variables, such as the participants’ level of study, faculty, age, gender, sexuality, Indigeneity, race, immigration status, living situation, and ability.

Following completion of the sociodemographic survey, participants from both study arms will also be asked to respond to additional battery of survey instruments, including the *Nature Exposure Scale (NES)*, the *Connectedness to Nature Scale (CNS)*, the *Satisfaction with Life Scale (SWLS)*, the *General Self-Efficacy Scale (GSE)*, the *Self-Rated Health Scale (SRH)*, and the *Pro-Environmental Behavior Scale (PEB)*. The NES provides a quantitative measure of an individual’s extent and quality of nature exposure (21). The CNS provides a quantitative measure of an individual’s emotional connection to nature (22). The SWLS provides a quantitative measure of an individual’s contentment with their day-to-day life (23). The GSE provides a quantitative measure of an individual’s optimistic beliefs about their ability to handle the demands of life (24). The SRH, taken from a longer 36-item instrument developed by Ware and Sherbourne (25), provides a quantitative measure of an individual’s self-perception of their overall health and wellbeing. Finally, the PEB provides a quantitative measure of an individual’s willingness to engage in pro-environmental behaviors and practices (26).

Participants in the intervention arm will be asked to complete the aforementioned battery of survey instruments at two points during the study: once before participating in the two-week-long *City Safari* intervention, and once at the end of the two-week-long intervention period. Likewise, participants in the non-intervention comparator arm will also be asked to complete the aforementioned battery of survey instruments at two distinct points during the study: once upon their enrolment, and once two weeks after completing their first.

During Phase B, data collection will primarily take the form of the modified photovoice methodology outlined by Sethi (27). Photovoice is a participatory, arts-based research method in which participants are provided with a topic, tasked with gathering photographs capturing their thoughts, and invited to reflect on the narratives emerging from their photographs (28). From its onset, photovoice was conceived as a way to challenge the power dynamics of traditional research paradigms, under which researchers assume positions of authority over their participants (28). The goal of photovoice is to empower participants by placing the tools of data collection directly in their hands, providing them with greater autonomy in the research process (28).

The first stage of photovoice will be integrated into the *City Safari* gameplay. The planned gameplay will already require the participants in the intervention arm to capture photographs in response to specific prompts. While the main motivation for participants to take photographs will be to score points in *City Safari*, their photographs may also provide insight into their thoughts and attitudes toward urban nature. It should be noted that because each of the five bingo cards consists of a five-by-five grid of squares, each participant may take as many as 125 photographs. To ensure that data can be reviewed and analyzed in a reasonable time frame, those included in Phase B will be asked to select just 5–7 of their photographs. This may encourage participants to select photographs with the greatest personal significance to them.

Under conventional approaches to photovoice, once participants have had the chance to gather photographs, they are typically invited to attend focus groups to share, discuss, and analyze the narratives emerging from their photographs. In contrast, the modified methodology created by Sethi (27) calls for participants to be interviewed separately through one-to-one conversations with the researcher. In this study, these one-to-one conversations will consist of 60–90-min semi-structured interviews, during which participants will be encouraged to guide the conversation as they see fit using their curated selection of photographs as prompts. Before implementation, the interview guides will undergo pilot testing with the aforementioned prototype focus groups. The results of these interviews will be used to develop a better understanding of the therapeutic potential of urban nature.

### Data analysis

To assess the impact of the *City Safari* gaming intervention on participants’ nature exposure and nature connectedness, pre-post changes in participant NES and CNS scores in the intervention arm will be compared with those in the non-intervention comparator arm using analysis of covariance (ANCOVA). In each ANCOVA model, the non-intervention comparator arm will serve as the baseline against which the intervention arm is compared. For the primary analyzes, intervention status will serve as the categorical independent variable, post-NES and CNS scores will serve as the dependent variable, and pre-NES and CNS scores will serve as the covariate. In addition to the pre-post changes in participants’ NES and CNS scores, exploratory ANCOVA models will be used to compare pre-post changes in participants’ SWLS, GSE, SRH, and PEB scores between the intervention and non-intervention groups. It should be acknowledged that while the two-week intervention period may not be enough time to observe a significant change in some of these variables—i.e., PEB, which is more ingrained and thus slower to shift—these variables may still be taken into consideration as covariates.

Before interpreting the results of the ANCOVA analysis, several steps will be taken to assess the assumptions underlying its use. First, to test the assumption that the regression slopes are homogeneous, i.e., parallel, each ANCOVA model will be rerun while including an interaction term between each covariate and independent variable (29). If the interaction term is found to be not significant, the regression lines in that model can be assumed to be homogeneous (29). Second, to test the assumption that the errors of the dependent variable are normally distributed, a Q-Q scatterplot will be generated to visually discern the degree to which the sample quantiles deviate from the theoretical quantiles. Third, to test the assumption of homoscedasticity, a scatterplot will be generated to visually discern the degree to which the residuals deviate from the predicted values. Fourth, to test for the presence of influential outliers, studentized residuals were calculated and plotted. Finally, to ensure the independence of error terms, participants in the intervention group were asked to complete their tasks independently of any other study participants.

Data collected from the modified photovoice process, namely the photographs and interview transcripts, will be analyzed through reflexive thematic analysis. As per Braun et al. (17), reflexive thematic analysis is a fully qualitative approach which recognizes the inseparability of the researcher’s personal position—their assumptions, values, lived experiences—from their interpretation of the data. While the researcher’s subjectivities must be clearly stated, the goal of reflexive thematic analysis is not to capture some sort of “objective truth”, but to identify patterns in meaning based on the researcher’s understanding of the data. The analytic process for this approach generally consists of the following six phases: (1) familiarization; (2) coding; (3) initial theme generation; (4) developing themes; (5) refining, defining, and naming themes; and (6) producing the report (17).

### Strategies to promote rigor or trustworthiness

In tandem with the reflexive thematic analysis, strategies will be employed to bolster the rigor and trustworthiness of the study findings. First, the lead author will maintain a reflexive journal, which will be used to document their written reflections on the research process. As per Olmos-Vega et al. (30), reflexive journaling can be a powerful tool for not only identifying but also critically evaluating how the researcher’s assumptions, biases, and attitudes might influence the conduct of the study.

“Member reflection” will also be employed to promote rigor and trustworthiness. This process should be distinguished from the related concept of “member checking”, a validation technique in which preliminary findings are presented to participants to assess their accuracy and resonance (30). Member reflection offers a more nuanced approach, in which study findings are acknowledged to be constructed in context. This allows for the understanding that participants might change perspectives between data collection and analysis (30). Member reflection not only allows participants to respond to the researcher’s interpretations of their earlier ideas but also gives them greater agency in how their words are disseminated to a wider audience.

The final strategy to promote rigor and trustworthiness will be reflexive dialogue. Throughout the study, the lead author will periodically share their emergent findings with other members of the research team. These periods of reflexive dialogue will provide the lead author with the opportunity to have their preliminary interpretations of the data challenged or corroborated. This additional scrutiny may allow the lead author to evaluate the extent to which their interpretations of the data align or conflict with those operating through distinct positionalities (30).

## Discussion

Given the importance of human-nature relations in a world increasingly defined by climate change, urbanization, and environmental disconnection, we anticipate that the results of this study may be relevant to research, policy, and practice. For starters, regarding research, the findings from this project will inform the writing of a minimum of three papers for peer-review publication as part of the lead author’s doctoral sandwich thesis. In addition to these three peer-reviewed publications, the findings from this study will also be disseminated to relevant academic audiences through paper and poster presentations at conferences in the fields of planetary health, human geography, and urban planning.

Outside of traditional academic channels, the findings from this study will also be disseminated to non-academic communities through: (1) the publication of policy briefs and community reports targeted at local governments, community organizations, and the general public; (2) the curation of multimedia art displays to share the narratives provided by participants through their photographs and interviews; and (3) the creation of informational webinars to share key insights with relevant community partners.

In addition to these strategies, and depending on the observed effectiveness of the *City Safari* game intervention, there may also be an opportunity to expand the scope of *City Safari* to a wider audience beyond the McMaster University student population. This process may involve collaborating with local organizations to tailor *City Safari’s gameplay* to meet the needs of their respective communities. Such collaborations will be necessary as our study, in its present form, will only comprise university students aged 18–25 years attending a single institution. The relative homogeneity of this sample may limit the external validity or transferability of the study findings to other populations.

Another limitation that must be acknowledged is the lack of blinding. Due to the nature of the study intervention, in which participants will know whether or not they have been assigned to take part in *City Safari*, it was not possible to blind participants to their group assignment. As such, this study design may introduce some confounding bias as participants alter their behaviors, either consciously or subconsciously, based on the knowledge that they are being observed or to align their actions with the perceived study expectations. Similarly, it must also be acknowledged that, as a result of knowing that they are in a study, some participants may be pushed towards quantity, rather than quality, in their picture taking. This may thin out the qualitative data provided by the pictures submitted by participants, which we have sought to address by asking participants to pick the 5–7 pictures which they deem to be the most personally significant.
